# Exploratory Study of Factors Influencing Job-Related Stress in Japanese Psychiatric Nurses

**DOI:** 10.1155/2015/805162

**Published:** 2015-04-02

**Authors:** Hironori Yada, Xi Lu, Hisamitsu Omori, Hiroshi Abe, Hisae Matsuo, Yasushi Ishida, Takahiko Katoh

**Affiliations:** ^1^Department of Nursing, Kumamoto Health Science University, 325 Izumi, Kumamoto, Kumamoto 861-5598, Japan; ^2^Department of Public Health, Faculty of Life Sciences, Kumamoto University, 1-1-1 Honjo, Kumamoto, Kumamoto 860-8556, Japan; ^3^Department of Psychiatry, Faculty of Medicine, University of Miyazaki, 5200 Kihara, Kiyotake, Miyazaki 889-1692, Japan

## Abstract

This study explored the factor structure of psychiatric nurses' job-related stress and examined the specificity of the related stressors using the job stressor scale of the Brief Job Stress Questionnaire (BJSQ). The stressor scale of the BJSQ was administered to 296 nurses and assistant nurses. Answers were examined statistically. Exploratory factor analysis was performed to identify factor structures; two factors (overload and job environment) were valid. Confirmatory factor analysis was conducted to examine the two-factor structure and found 11 items with factor loadings of >0.40 (model 1), 13 items with factor loadings from 0.30 to <0.40 (model 2), and 17 items with factor loadings from 0.20 to <0.30 (model 3) for one factor; model 1 demonstrated the highest goodness of fit. Then, we observed that the two-factor structure (model 1) showed a higher goodness of fit than the original six-factor structure. This differed from subscales based on general workers' job-related stressors, suggesting that the factor structure of psychiatric nurses' job-related stressors is specific. Further steps may be necessary to reduce job-related stress specifically related to overload including attention to many needs of patients and job environment including complex ethical dilemmas in psychiatric nursing.

## 1. Introduction

In recent years, because of the transition from hospital to community-based psychiatric care [1, 2], knowledge and skills of both community and hospital psychiatric nursing are necessary for treating psychiatric patients. Further, psychiatric patients are aging, leading to an increase in the number of patients with dementia; more than 40% of the total patient population is now over 65 years old [3]. Elderly patients often have physical complaints [4], and approximately 90% of elderly patients with dementia in psychiatric care have been reported to have physical complications that require treatment [5].

Caring for both the mental and physical health of the patient is especially important for psychiatric nurses; thus, their roles have continued to expand over the years, in association with increases in the mental health services supplied by psychiatric departments [6]. As a result of the expansion of their roles, several studies related to psychiatric nurses' job-related stress have been reported [7]. If psychiatric nurses' mental and physical health is not protected, psychiatric nurses often experience mental health disorders, which can have a negative influence on health care services [8]. In a report by Aronson [9], the employment turnover rate for psychiatric nurses was high and psychiatric nursing is regarded as a stressful occupation [10–12]. According to an investigation conducted by the Ministry of Health, Labour and Welfare in 2012, there are approximately 84,000 registered and assistant psychiatric nurses working in Japan [13]. Therefore, improving mental health care and working conditions for psychiatric nurses is an important industrial hygiene issue. However, to the best of our knowledge, few studies investigating the characteristics of psychiatric nurses' job-related stress have been conducted.

When the specific characteristics and structure of psychiatric nurses' job-related stressors are considered, they can be considered specific and unique compared to those of other workers [15]. Psychiatric nurses have an unusual working environment that includes locked ward entrances [16]; as a result, the potential for patient confrontation with the associated risk of both physical and mental danger [17], violence perpetrated by aggressive patients [18], and being required to seclude or restrain patients to prevent them from harming themselves or others [19] are potentially present. Therefore, the structures of job-related stressors experienced by psychiatric nurses potentially differ from those of other workers (Table 1).

From our previous study [17], three factors “workload,” “job control,” and “atmosphere” were extracted [17] as being important for job stressors, which was a result different from the factor structure of the job stressor obtained from general workers. However, our previous study had problems, including its small sample size (*n* = 36) and the unverified results of reliability and validity; therefore, our results could not be generalized for the larger population.

The aim of the present study was to confirm the specificity and validity of the factors that influence job-related stressors in psychiatric nurses.

## 2. Materials and Methods

### 2.1. Participants

Anonymous self-administered questionnaires were sent via mail to 385 nurses and assistant nurses in six psychiatric hospitals between November 17, 2009, and December 21, 2009. Participants were informed of the aims of the investigation, and their written consent was obtained. The study protocol was approved by the Ethics Committee of Kumamoto University Graduate School of Life Sciences.

### 2.2. Questionnaire

The job stressor scale in the Brief Job Stress Questionnaire (BJSQ) was used to reveal participants' job-related stressor levels [20]. The scores on the scale range from 1 to 4, with higher scores indicating a higher job-related stressor level. The job stressor scale measures quantitative overload (items 1–3), mental demand (items 4–6), physical workload (item 7), job control (items 8–10), utilization of techniques (item 11), interpersonal relations (items 12–14), work environment (item 15), fit to the job (item 16), and reward for the work (item 17).

Then, the National Institute for Occupational Safety and Health job stress model proposes that stress reactions are affected by job stressors [21]. In this study, the predictive validity of the factor structure is validated by examining the relation between the stress factors and the experienced stress. The stress reaction scale in the Brief Job Stress Questionnaire (BJSQ) [20] was used to measure stress reactions. The stress reaction scale measures psychological and physical stress reactions. The psychological stress reaction scale assesses lack of vigour (items 1–3), irritability (items 4–6), fatigue (items 7–9), anxiety (items 10–12), and depressed mood (items 13–18). Physical stress reactions were assessed with a somatic symptoms subscale (items 19–29). These scales have been used in a number of recent job-related stress studies [22–30] and are useful for the evaluation of job related stress in different fields. Job-related stress was analysed in individuals who work in many industries during the development of the BJSQ [14]. The reliability and validity of the scale have been verified [14]. A six-factor structure was suggested for many industries [14]. Table 1 shows the six-factor structure. Translation of sentences from Japanese to English in the item content of the “job stressor” scale used phrases from a previous study [31].

### 2.3. Statistical Analysis

SPSS version 17.0 software package for Windows (SPSS, Chicago, IL, USA) was used for item analysis, extraction of factors, and calculation of internal consistency and cross-validation (split-half method by random sampling), and predictive validity. Amos version 17.0 software package for Windows (AMOS, Chicago, IL, USA) was used to determine the compatibility of the model.

## 3. Results

### 3.1. Questionnaire Response Rate

Three hundred forty-seven psychiatric and assistant nurses responded to the mailed questionnaires. Among the respondents, 296 subjects who gave their informed consent of the investigation were accepted as subjects for analysis (effective response rate: 85.3%). The mean age of the participants was 42.5 ± 11.1 years; 94 were males (31.8%), 193 were females (65.2%), and nine did not reveal their gender (3.0%). Forty-one participants were managers (head or chief nurse; 13.9%), 228 were nonmanagers (77.0%), and 27 did not reveal this information (9.1%). With regard to qualifications, 196 participants were nurses (66.2%), 90 were assistant nurses (30.4%), and 10 did not reveal this information (3.4%). A total of 199 participants (67.2%) had experience in other departments, 87 did not (29.4%), and 10 did not reveal this information (3.4%). The mean number of years' psychiatry department experience was 13.3 ± 10.5 years. The results are shown in Table 2.

### 3.2. Results of Item Analysis

The number of missing values for each item was 0–2, which we judged to be small [15]. The mean item-score for the missing values was substituted in the statistical analysis [15]. None of the items had a ceiling or floor effect in M ± 1S.D.; therefore, all items were included in the subsequent statistical analysis.

### 3.3. Results of Factor Extraction, Internal Consistency, Cross-Validation, and Predictive Validity Calculation

The factor structure of the participants' job-related stressors obtained with the job stressor scale [20] was identified using exploratory factor analysis (EFA). In the process of conducting the EFA, the Kaiser-Meyer-Olkin (KMO) measure of sampling adequacy and Bartlett's test of sphericity (*X* ^²^) were confirmed. The maximum likelihood method was used for factor extraction; promax rotation was also conducted. A scree test [32] was used to determine the number of factors involved. The KMO measure of sampling adequacy was 0.740, indicating that it was appropriate to analyse the data using EFA [33]. Bartlett's test of sphericity (*X* ^²^) was 1211.36 (df = 136) *P* < 0.001, indicating that it was an acceptable value. The attenuation situation of the five eigenvalues that were higher than 1.0 was 3.44, 2.80, 1.36, 1.14, and 1.08, and the number of factors was valid in factor analysis. Cumulative contribution rate was 36.66% for two factors. The overload factor, including items that were related to quantitative overload, mental demand, and physical workload, and the job environment factor, including items related to the surrounding environment, were extracted in the EFA. The results are shown in Table 3. Items 8 and 9 showed factor loadings ranging from 0.30 to <0.40 for one factor, and items 11, 12, 13, and 15 showed factor loadings ranging from 0.20 to <0.30 for one factor. Cronbach's *α* coefficients in factors in which items with a low factor loading were deleted were 0.79 (first factor: overload) and 0.71 (second factor: job environment). For cross-validation, Pearson's correlation coefficient was 0.96 (*P* < 0.01) in the overload factor, indicating a strong correlation. Pearson's correlation coefficient was 0.99 (*P* < 0.01) in the job environment factor, indicating a strong correlation. Tests of predictive validity (Pearson's correlation analysis) also confirmed positive correlations within a significance of *P* < 0.01 for almost all of the associations between stress reaction scale and the overload factor and the job environment factor (Table 4).

### 3.4. Goodness of Model Fit for Each Factor Structure

Confirmatory factor analysis (CFA) was conducted to explore the valid factor structure of psychiatric nurses' job-related stressors. Goodness of fit of the model was confirmed and compared between the two-factor structure that was calculated by EFA in this study and the six-factor structure. Goodness of model fit was confirmed by six indices (*χ* ^2^/df ratio; GFI: goodness-of-fit index; AGFI: adjusted goodness-of-fit index; CFI: comparative fit index; RMSEA: root mean square error of approximation; AIC: Akaike information criterion) [34] and compared in the two-factor structure that was calculated using EFA. If the estimated error distribution showed a negative solution, the variance was set to zero [35]. In addition, when the two-factor structure was used for CFA, items 8, 9, 11, 12, 13, and 15 showed factor loading of <0.40 for one factor. CFA was then conducted in the three patterns (model 1: items included factor loading of ≥0.40 for one factor; model 2: items included factor loading of ≥0.30 for one factor; and model 3: items included factor loading of ≥0.20 for one factor) and each model in the two-factor structure was examined for goodness of fit [36].  Table 5 shows the results. With regard to the goodness of fit of the model in the two-factor structure, model 1 was *χ* ^2^/df ratio = 2.49 (107.63/43, *P* < 0.01), GFI = 0.94, AGFI = 0.91, CFI = 0.92, RMSEA = 0.07, and AIC = 153.63; model 2 was *χ* ^2^/df ratio = 3.93 (251.83/64, *P* < 0.01), GFI = 0.88, AGFI = 0.83, CFI = 0.81, RMSEA = 0.10, and AIC = 305.83; and model 3 was *χ* ^2^/df ratio = 2.98 (345.73/116, *P* < 0.01), GFI = 0.88, AGFI = 0.84, CFI = 0.79, RMSEA = 0.08, and AIC = 419.73. In contrast, the goodness of fit of the model in the six-factor structure model was *χ* ^2^/df ratio = 2.23 (229.27/103, *P* < 0.01), GFI = 0.92, AGFI = 0.89, CFI = 0.89, RMSEA = 0.06, and AIC = 329.27.

## 4. Discussion

The aim of this study was to confirm specificity and validity of the factor structure of psychiatric nurses' job-related stressors using 17 items on the job stressor scale of the BJSQ [20]. The two-factor structure with 11 items was different from the six-factor structure for industries [14] and the most valid one. The following is a discussion of the results.

### 4.1. Examination Related to the Adoption of a Specific Factor Structure for Psychiatric Nurses' Stressors

**Figure 1:**
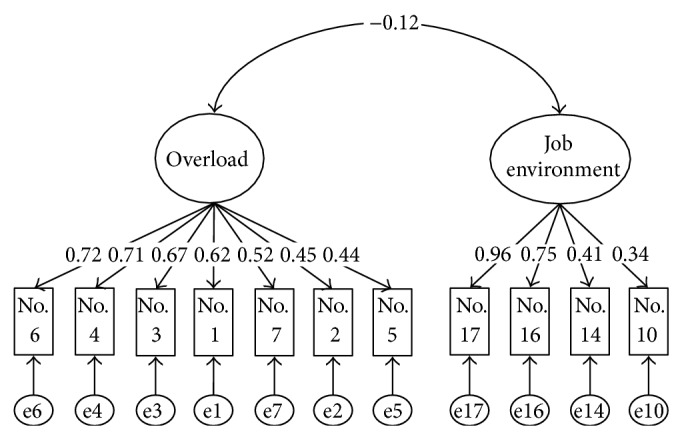
Two-factor structure; model 1. *χ* ^2^/df ratio = 2.49 (107.63/43, *P* < 0.01), GFI = 0.94, AGFI = 0.91, CFI = 0.92, RMSEA = 0.07.

The two-factor structures (model 1: items included factor loading of ≥0.40 for one factor; model 2: items included factor loading of ≥0.30 for one factor; model 3: items included factor loading of ≥0.20 for one factor) and the six-factor structure were analysed by CFA. In model 1, the AIC was superior to the AIC for models 2 and 3 and six-factor structure model, and while the other indices (GFI, AGFI, CFI, RMSEA) were also better, they were similar to the general standard (*χ* ^2^/df ratio < 3, GFI > 0.90, AGFI > 0.90, CFI > 0.90, RMSEA < 0.08) [37]. The *χ* ^2^/df ratio for model 1 was slightly poorer than for the six-factor structure. However, the AIC in model 1 was better than in the six-factor structure. When all of the results were compared, model 1 (two-factor structure with 11 items) was the most valid factor structure. Cronbach's *α* coefficients were 0.79 and 0.71 for the first (overload) and second (job environment) factors, respectively, as shown in Table 3. For internal consistency confirmation, Cronbach's *α* of >0.6 is generally preferred [38]; this was exceeded by model 1 in this study. For cross-validation, correlation coefficients were 0.96 and 0.99 for the overload and job environment factors. Tests of predictive validity (Pearson's correlation analysis) also confirmed positive correlations within a significance of *P* < 0.01 for almost all of the associations between stress reaction scale and the overload factor and the job environment factor (Table 4). We therefore deemed two-factor structure to have cross-validity.  Figure 1 shows the two-factor structure (model 1) that was finally adopted.

### 4.2. Examination Related to the Specificity of Psychiatric Nurses' Job-Related Stressors

As shown in Table 5, the two-factor structure with 11 items was valid in this study, and the job stressor scale [20] factor structure calculated in this study was considered partially different from existing subscales. In a previous study conducted by Shimomitsu and Haratani [14], the items (1–7) related to overload (quantitative overload, mental demand, and physical workload) were divided chiefly into three areas representing general workers' job-related stressor factor structure, whereas items (1–7) that related to overload were integrated into one factor in the present psychiatric nurses' job-related stressor factor structure. Therefore, considering the first factor in model 1 in this study and the existing factor structure from the previous study [14], it could be reasonable to assume that physical overload causes mental overload and physical workload. When psychiatric care was surveyed in recent years, it was observed that psychiatric patients were aging [3]. More than half of psychiatric patients suffer from internal diseases [39], and elderly psychiatric patients have multiple diseases and nursing needs, suffering from both mental illness and physical complications [4]. Psychiatric nurses have to pay attention to many medical conditions of patients. Moreover, more than 80% of elderly patients with dementia in psychiatric departments need care that includes meals and help with bathing, toileting, and dressing [5]. In a psychiatric nursing setting, caring for both the mental and physical needs of patients is required. Therefore, psychiatric nurses may be not adjusted to the increased care demands; they must consider changes in patients' functional ability in addition to experiencing increasing quantitative overload and physical workloads, which are necessary to adequately support activities of daily living (ADL) and treat elderly patients' physical symptoms. Items (1–3) related to quantitative overload, mental demand (4–6), and physical workload (7) were integrated into a single factor, and psychiatric nurses' job-related stressor structure was considered to be specific.

The job environment factor examined in this study comprised items related to the nurses' working environment, including rewards for the work (item 17), fit to the job (item 16), atmosphere (item 14), and job control (item 10). Patients who require psychiatric nursing have significant problems, including long-term hospitalization and frequent disease relapse [40]. Therefore, unlike in many other nursing fields, psychiatric nurses tend to have difficulty obtaining positive outcomes from their work; this may influence the sense of reward they experience, which is thought to be related to the job environment factor. In addition, Japanese psychiatric nurses are subject to the restrictions of the “Act on Mental Health and Welfare for the Mentally Disabled” in addition to other medical laws [41, 42]. Therefore, psychiatric nurses are required to attempt to protect patients and others from danger, whilst limiting any action they take against patients [18]. As a result, they face complex ethical dilemmas [43] and confrontational attitudes toward psychiatric nursing [17], both of which are associated with motivation for working in the field and job control, which is thought to be related to the Job Environment factor.

## 5. Study Limitation

In this study, the factor structure for job-related stressor was compared between psychiatric nurses and general workers, but not between psychiatric nurses and nurses from other departments. Thus, it is unclear whether the job-related stressors affecting the psychiatric nurses are unique to them, or whether nurses in other departments face similar job-related stressors. Future studies should address this issue.

## 6. Conclusions

When the stressors experienced by psychiatric nurses were evaluated using the BJSQ job stressor scale [20], model 1, with a two-factor structure and 11 items, was most valid, which differed from existing subscales that were standardized based on stressor in general workers. That is, psychiatric nurses' stress was primarily influenced by the overload including attention to many needs of patients and job environment including complex ethical dilemmas toward psychiatric nursing, and this was specific to their occupation. Our findings suggest that, as a provision for protecting psychiatric nurses, further steps should be taken to reduce job-related stressor specifically related to overload, as calculated in this study, by reviewing role-differentiation in the standard working conditions for psychiatric nursing and nursing staff. In addition, with regard to job-related stress related to job environment, as described in this study, opportunities for learning and intervention in psychiatric nursing should be developed. It is also necessary to improve work environments by promoting understanding of the unique stressors and difficult situations that have an impact on psychiatric nurses.

## Figures and Tables

**Table 1:** Job stress factors structures in the previous study.

Item number	Content of items	*F*1	*F*2	*F*3	*F*4	*F*5	*F*6
Number 1	Large amount of work	0.39	0.10	**0.76**	0.00	0.05	0.10
Number 2	Not enough time to get job done	0.23	0.15	**0.83**	0.07	0.00	0.04
Number 3	Requires working hard	**0.62**	0.05	**0.42**	0.05	0.01	0.20
Number 4	Requires concentration	**0.82**	0.03	0.04	0.05	0.05	0.17
Number 5	Complex job which requires high level of knowledge and skills	**0.68**	0.15	0.15	0.05	0.24	0.18
Number 6	Requires always thinking about job during work hours	**0.72**	0.01	0.27	0.06	0.02	0.02
Number 7	A lot of physical work	0.16	0.05	0.05	0.11	0.01	**0.78**
Number 8	Can work at own pace	0.16	0.05	**0.40**	**0.71**	0.10	0.02
Number 9	Can decide order and ways to do jobs	0.01	0.02	0.02	**0.86**	0.08	0.13
Number 10	Can express opinion of worksite policy	0.06	0.26	0.25	**0.61**	0.22	0.15
Number 11	Requires less skill and expertise than I have	0.12	0.01	0.02	0.11	**0.65**	0.35
Number 12	Interpersonal conflict within a workgroup	0.05	**0.76**	0.16	0.04	0.12	0.11
Number 13	Conflicts with other workgroups	0.01	**0.76**	0.16	0.01	0.05	0.10
Number 14	Friendly atmosphere in a workgroup	0.12	**0.63**	0.15	0.21	0.24	0.05
Number 15	Not good to workplace environments	0.01	0.30	0.00	0.13	0.05	**0.62**
Number 16	Contents of job that suit oneself	0.01	0.19	0.03	0.26	**0.79**	0.10
Number 17	Rewardable job	0.14	0.26	0.08	0.23	**0.75**	0.07

Extract from the previous study [14].

*F*: factor.

Factor loadings with absolute values ≧0.40 are in boldface.

**Table 2:** Characteristics of subjects (*N* = 296).

	Mean	S.D.	*N*	%
Age (years)	42.5	11.1		
Years of experience in psychiatry department	13.3	10.5		
Gender				
Male			94	31.8%
Female			193	65.2%
Unanswered			9	3.0%
Job position				
Manager (Head Nurse of Chief Nurse)			41	13.9%
Nonmanager			228	77.0%
Unanswered			27	9.1%
Qualification				
Nurse			196	66.2%
Assistant nurse			90	30.4%
Unanswered			10	3.4%
Experience in other departments				
Yes			199	67.2%
No			87	29.4%
Unanswered			10	3.4%

**Table 3:** Two-factor structure in the present study.

Item number	Content of items	*F*1	*F*2	M	S.D.	Communality
*F*1: overload (Cronbach's *α* coefficient = 0.79); 7 items						
6	Requires always thinking about job during work hours	**0.71**	0.05	2.94	0.78	0.51
4	Requires concentration	**0.69**	−0.01	3.23	0.69	0.48
3	Requires working hard	**0.68**	−0.04	3.17	0.73	0.46
1	Large amount of work	**0.62**	0.14	2.94	0.76	0.41
7	A lot of physical work	**0.51**	0.06	2.94	0.88	0.27
2	Not enough time to get job done	**0.46**	0.08	2.44	0.89	0.22
5	Complex job which requires high level of knowledge and skills	**0.42**	−0.02	2.81	0.78	0.17

*F*2: job environment (Cronbach's *α* coefficient = 0.71); 4 items						
17	Rewardable job	−0.20	**0.84**	2.21	0.83	0.74
16	Contents of job that suit oneself	0.07	**0.80**	2.32	0.74	0.65
14	Friendly atmosphere in a workgroup	0.02	**0.45**	2.03	0.78	0.20
10	Can express opinion of worksite policy	0.09	**0.42**	2.62	0.74	0.19
8	Can work at own pace	0.25	0.33	2.71	0.77	0.17
9	Can decide order and ways to do jobs	0.15	0.30	2.32	0.78	0.11
11	Requires less skill and expertise than I have	−0.02	0.29	2.30	0.70	0.08
15	Not good to workplace environments	0.11	0.28	2.58	0.96	0.09
13	Conflicts with other workgroup	0.18	0.28	2.16	0.79	0.11
12	Interpersonal conflict within a workgroup	0.10	0.24	2.63	0.76	0.07

	Factor correlation	1				
		0.04	1			

Factor loadings with absolute values ≥0.40 are in boldface.

*F*: factor.

M: mean.

S.D.: standard deviation.

**Table 4:** Two-factor and stress reactions correlations.

	Lack of vigor	Irritability	Fatigue	Anxiety	Depressed mood	Somatic symptoms
Overload	0.10	0.26^∗^	0.37^∗^	0.45^∗^	0.28^∗^	0.26^∗^
Job environment	0.52^∗^	0.35^∗^	0.33^∗^	0.16^∗^	0.36^∗^	0.23^∗^

^*^ *P* < 0.01.

**Table 5:** The goodness of fit in models.

	*χ* ^2^/df ratio	GFI	AGFI	CFI	RMSEA	AIC
Model 1: including items of factor loading (absolute value) 0.40 or more^†^	2.49 (107.63/43^∗^)	0.94	0.91	0.92	0.07	153.63
Model 2: including items of factor loading (absolute value) 0.30 or more	3.93 (251.83/64^∗^)	0.88	0.83	0.81	0.10	305.83
Model 3: including items of factor loading (absolute value) 0.20 or more	2.98 (345/116^∗^)	0.88	0.84	0.79	0.08	419.73
Original six-factor structure	2.23 (229.27/103^∗^)	0.92	0.89	0.89	0.07	329.27

^†^Model 1 was adopted by confirmatory factor analysis.

^∗^ *P* < 0.01.

## References

[1] Fioritti A., Giaccotto L., Melega V. (1997). Choking incidents among psychiatric patients: retrospective analysis of thirty-one cases from the West Bologna psychiatric wards. *Canadian Journal of Psychiatry*.

[2] Read A. (2009). Psychiatric deinstitutionalization in BC: negative consequences and possible solutions. *UBC Medical Journal*.

[3] Tanioka T., Kataoka M., Yasuhara Y., Miyagawa M., Ueta I. (2011). The role of nurse administrators and managers in quality psychiatric care. *The Journal of Medical Investigation*.

[4] Ukai K., Mizuno Y. (2009). Physical complications for elderly inpatients with senile dementia in the Imaise Branch of Ichinomiya City Hospital. *Psychogeriatrics*.

[5] http://www.mhlw.go.jp/stf/shingi/2r9852000000xcat-att/2r9852000000xcgs.pdf.

[6] Nolan P., Carr N., Doran M. (2004). Nurse prescribing: the experiences of psychiatric nurses in the United States. *Nursing Standard*.

[7] Yada H., Omori H., Funakoshj Y., Katoh T. (2010). Current state of research on occupational stress of psychiatric nurses and insight into its future. *Journal of UOEH*.

[8] Dawkins J. E., Depp F. C., Selzer N. E. (1985). Stress & the psychiatric nurse. *Journal of Psychosocial Nursing and Mental Health Services*.

[9] Aronson K. R. (2005). Job satisfaction of nurses who work in private psychiatric hospitals. *Psychiatric Services*.

[10] Melchior M. E. W., van den Berg A. A., Halfens R., Abu-Saad H. H., Philipsen H., Gassman P. (1997). Burnout and the work environment of nurses in psychiatric long-stay care settings. *Social Psychiatry and Psychiatric Epidemiology*.

[11] Shen H.-C., Cheng Y., Tsai P.-J., Lee S.-H. S., Guo Y. L. (2005). Occupational stress in nurses in psychiatric institutions in Taiwan. *Journal of Occupational Health*.

[12] Yang M.-S., Pan S.-M., Yang M.-J. (2004). Job strain and minor psychiatric morbidity among hospital nurses in southern Taiwan. *Psychiatry and Clinical Neurosciences*.

[13] http://www.mhlw.go.jp/stf/shingi/2r985200000264pr-att/2r985200000264x9.pdf.

[14] Shimomitsu T., Haratani T. (2000). *A Research Report Relating to Stress in the Workplace and Its Impact on Worker's Health*.

[15] Yada H., Abe H., Funakoshi Y. (2011). Development of the Psychiatric Nurse Job Stressor Scale (PNJSS). *Psychiatry and Clinical Neurosciences*.

[16] Haglund K., Von Essen L. (2005). Locked entrance doors at psychiatric wards—advantages and disadvantages according to voluntarily admitted patients. *Nordic Journal of Psychiatry*.

[17] Yada H., Abe H., Omori H., Ishida Y., Katoh T. (2009). Stressor among nurses in a psychiatric department—comparison between acute and recuperation wards. *Journal of UOEH*.

[18] Nijman H., Bowers L., Oud N., Jansen G. (2005). Psychiatric nurses' experiences with inpatient aggression. *Aggressive Behavior*.

[19] Metzner J. L., Tardiff K., Lion J. (2007). Resource document on the use of restraint and seclusion in correctional mental health care. *Journal of the American Academy of Psychiatry and the Law*.

[20] Shimomitsu T., Odagiri Y. (2004). The brief job stress questionnaire. *Sangyo Seishin Hoken*.

[21] Hurrell J. J., McLaney M. A. (1988). Exposure to job stress—a new psychometric instrument. *Scandinavian Journal of Work, Environment & Health*.

[22] Harada H., Suwazono Y., Sakata K. (2005). Three-shift system increases job-related stress in Japanese workers. *Journal of Occupational Health*.

[23] Kawada T., Otsuka T. (2011). Relationship between job stress, occupational position and job satisfaction using a brief job stress questionnaire (BJSQ). *Work*.

[24] Kawakami N., Takao S., Kobayashi Y., Tsutsumi A. (2006). Effects of web-based supervisor training on job stressors and psychological distress among workers: a workplace-based randomized controlled trial. *Journal of Occupational Health*.

[25] Kawano Y. (2008). Association of job-related stress factors with psychological and somatic symptoms among Japanese hospital nurses: effect of departmental environment in acute care hospitals. *Journal of Occupational Health*.

[26] Mineyama S., Tsutsumi A., Takao S., Nishiuchi K., Kawakami N. (2007). Supervisors' attitudes and skills for active listening with regard to working conditions and psychological stress reactions among subordinate workers. *Journal of Occupational Health*.

[27] Muto S., Muto T., Seo A., Yoshida T., Taoda K., Watanabe M. (2007). Job stressors and job stress among teachers engaged in nursing activity. *Industrial Health*.

[28] Tahara H., Yamada T., Nagafuchi K. (2009). Development of a work improvement checklist for occupational mental health focused on requests from workers. *Journal of Occupational Health*.

[29] Umehara K., Ohya Y., Kawakami N., Tsutsumi A., Fujimura M. (2007). Association of work-related factors with psychosocial job stressors and psychosomatic symptoms among Japanese pediatricians. *Journal of Occupational Health*.

[30] Urakawa K., Yokoyama K., Itoh H. (2012). Sense of coherence is associated with reduced psychological responses to job stressors among Japanese factory workers. *BMC Research Notes*.

[31] Kazunori I., Hiroyuki T., Tatsuji Y. (2010). The effects of a mental health training program for manufacturing company managers. *Journal of UOEH*.

[32] Cattell R. B. (1966). The scree test for the number of factors. *Multivariate Behavioral Research*.

[33] Kaiser H. F. (1974). An index of factorial simplicity. *Psychometrika*.

[34] Igarashi H., Kikuchi H., Kano R. (2009). The inventory of personality organisation: its psychometric properties among student and clinical populations in Japan. *Annals of General Psychiatry*.

[35] Yamamoto K., Onodera T. (2006). *Covariance Structure Analysis and the Case of Analysis by Amos*.

[36] Matsudaira T., Igarashi H., Kikuchi H. (2009). Factor structure of the hospital anxiety and depression scale in Japanese psychiatric outpatient and student populations. *Health and Quality of Life Outcomes*.

[37] Ouyang Y. (2009). The mediating effects of job stress and job involvement under job instability: banking service personnel of Taiwan as an example. *Journal of Money, Investment and Banking*.

[38] George D., Mallery P. (2003). *SPSS for Windows Step by Step: A Simple Guide and Reference. 11.0 Update*.

[39] Fogel B. S. (1985). A psychiatric unit becomes a psychiatric-medical unit: administrative and clinical implications. *General Hospital Psychiatry*.

[40] Suzuki Y., Yasumura S., Fukao A., Otani K. (2003). Associated factors of rehospitalization among schizophrenic patients. *Psychiatry and Clinical Neurosciences*.

[41] Asai K. (1998). From mental health law to mental health and welfare law. *Psychiatry and Clinical Neurosciences*.

[42] Tateno M., Sugiura K., Uehara K. (2009). Attitude of young psychiatrists toward coercive measures in psychiatry: a case vignette study in Japan. *International Journal of Mental Health Systems*.

[43] Kontio R., Välimäki M., Putkonen H., Kuosmanen L., Scott A., Joffe G. (2010). Patient restrictions: are there ethical alternatives to seclusion and restraint?. *Nursing Ethics*.

